# *Alu* RNA Modulates the Expression of Cell Cycle Genes in Human Fibroblasts

**DOI:** 10.3390/ijms20133315

**Published:** 2019-07-05

**Authors:** Simona Cantarella, Davide Carnevali, Marco Morselli, Anastasia Conti, Matteo Pellegrini, Barbara Montanini, Giorgio Dieci

**Affiliations:** 1 Department of Chemistry, Life Sciences and Environmental Sustainability, University of Parma, 43124 Parma, Italy; 2 Molecular, Cell and Developmental Biology Department, University of California, Los Angeles, Los Angeles, CA 90095, USA; 3 San Raffaele Telethon Institute for Gene Therapy (SR-Tiget), IRCCS San Raffaele Scientific Institute, 20132 Milan, Italy

**Keywords:** *Alu* retrotransposons, cell cycle, non-coding RNA

## Abstract

*Alu* retroelements, whose retrotransposition requires prior transcription by RNA polymerase III to generate *Alu* RNAs, represent the most numerous non-coding RNA (ncRNA) gene family in the human genome. *Alu* transcription is generally kept to extremely low levels by tight epigenetic silencing, but it has been reported to increase under different types of cell perturbation, such as viral infection and cancer. *Alu* RNAs, being able to act as gene expression modulators, may be directly involved in the mechanisms determining cellular behavior in such perturbed states. To directly address the regulatory potential of *Alu* RNAs, we generated IMR90 fibroblasts and HeLa cell lines stably overexpressing two slightly different *Alu* RNAs, and analyzed genome-wide the expression changes of protein-coding genes through RNA-sequencing. Among the genes that were upregulated or downregulated in response to *Alu* overexpression in IMR90, but not in HeLa cells, we found a highly significant enrichment of pathways involved in cell cycle progression and mitotic entry. Accordingly, *Alu* overexpression was found to promote transition from G1 to S phase, as revealed by flow cytometry. Therefore, increased *Alu* RNA may contribute to sustained cell proliferation, which is an important factor of cancer development and progression.

## 1. Introduction

**Figure 1:**
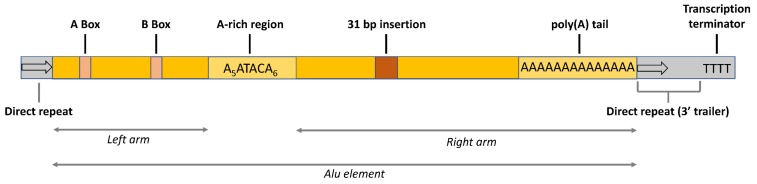
Sequence features of a consensus *Alu* element. The left arm harbors the internal Pol III promoter, composed of the A Box and B Box. The right arm has a 31-bp insertion. The two arms are separated by an A-rich region and the entire *Alu* element ends with a poly(A) tail. Grey indicates the genomic repeats that originate from the retrotransposition event and the 3′ trailer sequence between the poly(A) tail and the canonical terminator (at least four Ts).

As much as 10% of the human genome is composed of *Alu* elements, which are highly repetitive retrotransposons belonging to the class of the short interspersed nuclear elements (SINEs), and count for a total of more than one million copies in the whole set of the human chromosomes [1]. It is thought that *Alu* sequences originated 65 million years ago from the retrotransposition of the 7SL RNA, an event that coincides with the radiation of primates [2,3]. During their amplification, *Alu* sequences accumulated base substitutions that led to their classification into three subfamilies: the oldest and the intermediate age *AluJ* and *AluS* subfamilies, which are no longer retrotranspositionally active, and the youngest *AluY* subfamily, which is still able to retrotranspose in germ cell lines [4]. *Alu* retrotransposition depends on non-LTR retroelements LINE-1 (L1)-encoded ORF1p and ORF2p proteins, in order to reintegrate in the genome via a target-primed reverse transcription mechanism. The exact process used by *Alu* retroelements to target the genome is unknown, but there is strong evidence that *Alu* retrotransposition is biased towards gene-rich regions [5], both at intergenic loci and at intragenic positions. Possible targets of gene regions are represented by 5′ and 3′ untranslated regions (5′ UTRs and 3′ UTRs) and by introns of protein-coding genes, with a non-random distribution according to gene functional categories [6]. The consensus *Alu* sequence is about 300 nucleotides in length and is thought to derive from the head to tail fusion of two distinct 7SL RNA genes [7]. The dimeric *Alu* sequence is composed of a left arm, which harbors the A and B boxes derived from the 7SL RNA polymerase III (Pol III) promoter, and a right arm, which has an additional 31-bp insertion. The left and the right arms are separated by an intermediate A-rich consensus sequence (A_5_TACA_6_) and the element ends with a relatively long poly(A) tail (Figure 1). The 3′-trailer region between the *Alu* poly(A) tail and the first encountered termination signal (a run of at least four Ts or a T-rich non-canonical terminator) is unique to each individual *Alu* RNA. The potential mutagenic effect that could arise from the frequent insertion of *Alu* elements during their amplification in primates, *Alu’s* highly repetitive nature, the lack of a protein-coding potential, and low levels of transcription mainly due to epigenetic silencing, led to *Alu* elements being referred to as “parasites” of the human genome. However, this hypothesis does not explain the lack of negative selection during evolution, or why *Alu* elements are maintained at such a high copy number in the human genome. These features instead suggest the possibility that *Alus* could play important regulatory roles. Indeed, currently there is evidence for the involvement of *Alus* in a multitude of gene regulatory processes through *cis* and *trans* mechanisms. *Cis* mechanisms rely on (i) the insertion of new transcription factor binding sites that are present in *Alu* sequences, influencing the expression of genes involved in differentiation and development [8], (ii) the influence of intragenic *Alus* on pre-mRNA splicing [9], (iii) the evolution of *Alu* elements into new enhancers, influencing the expression of genes that are far away in the genome [10], and (iv) genomic rearrangements that could arise from *Alu* insertion, which usually lead to the development of disease [11]. *Alu* sequences can also influence gene regulation and other processes in *trans*, due to the ability of *Alu* transcripts to (i) bind RNA polymerase II (Pol II) and inhibit transcription initiation [12], (ii) regulate mRNA nuclear export via a p54nrb protein (also known as “Nono”) [13,14], (iii) influence translation by binding to the SRP9/14 subunit of the signal recognition particle (SRP) [15], and (iv) activate the NLRP3 inflammasome [16]. Additionally, *Alu* RNA sequences embedded in longer transcripts may exert other effects, such as the induction of ADAR-dependent RNA editing of mRNAs that carry *Alu* inverted repeats [17,18], the alteration of translation efficiency by base-pairing of inverted *Alu* repeats in the 3′ UTR of mRNA genes [19], the stimulation of circRNA biogenesis by backsplicing [20,21], and the control of nuclear localization of long non-coding RNAs [22]. It is known that, in physiological cell conditions, *Alu* elements are epigenetically silenced [23] and their expression is dramatically increased following different types of cell stress, such as virus infection [24], heat shock [25], cancer progression [26], epithelial to mesenchymal transition [27], and the age-related macular degeneration [28], supporting the hypothesis that *Alu* RNA may play important roles in both physiological and pathological contexts. However, it is not clear if *Alu* overexpression functions to overcome the stress condition, or if the increase in *Alu* RNA is a mere functionally irrelevant consequence of a global change in genome transcription. In spite of the different functions that *Alu* RNAs may have in gene regulation and other processes, a clear and comprehensive picture of the impact of *Alu* RNAs on cell growth and proliferation is still lacking. Recently, Di Ruocco et al. [27] found no perturbation of cell cycle distribution of colorectal cancer cells in response to the transient transfection of a synthetic *Alu*. On the other hand, Sakamoto et al. [29] previously found that HeLa cell growth was inhibited after the transient transfection of endogenous and synthetic *Alu* sequences, and Baryakin et al. [30] showed that the transient transfection of an *Alu*-RNA analogue suppresses cell proliferation and induces apoptosis of breast cancer cells. In line with this, Hu et al. [31] reported an inhibition of stem cell proliferation after the microinjection of an *AluSx* overexpressing vector in Ntera2 cells; Morales-Hernandèz et al. [32] showed that the transient transfection of the NANOG *Alu* sequence (localized upstream of the NANOG gene) repressed the expression of the pluripotency genes OCT4 and NANOG in Ntera cells; and Castelnuovo et al. [33] reported a differentiation-promoting effect of an *Alu*-like RNA in neuroblastoma cells. These observations suggest that *Alu* RNA might exert an inhibitory effect on cell proliferation; it has to be noted, however, that in these studies *Alu* RNAs were overexpressed through transient transfection and their effects were analyzed in tumor or stem cells, in which the expression of cell cycle genes is already skewed toward proliferation. 

As an additional step to understand the relationship between *Alu* expression and cell proliferation, the present study shows that the stable overexpression of two *Alu* elements of the *AluS* subfamily shows no effect in HeLa cells, but that it causes a significant upregulation of cell cycle-promoting genes in primary human fibroblasts, suggesting a function for *Alu* elements in regulating the cell cycle of normal cells.

## 2. Results

### 2.1. Alu Sequences, Vectors, and Cell Lines for Overexpression

**Figure 2:**
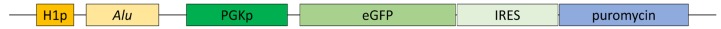
Schematic representation of the DNA inserted by the lentivirus vector used to generate IMR90 and HeLa cells that stably overexpress *Alu* sequences. The Pol III H1 promoter (H1p) controls the expression of *AluSq2*, *AluSx*, or a Control RNA. An empty vector was used as negative control. The eGFP gene and the gene coding for puromycin resistance are inserted under the control of the PGK gene promoter (PGKp).

The main questions we addressed in this study were whether and how an increased level of *Alu* RNA, derived from transcription of one or more *Alu* elements, affected the protein-coding transcriptome in human cell lines. HeLa cells were chosen as a model for tumor cells and IMR90 primary fetal lung fibroblast as a model for non-tumor, normal human cells. *Alu* transcripts are generally extremely similar in their sequence and thus expected to induce very similar functional consequences upon overexpression. We selected two individual *Alu* elements that we found reproducibly expressed in both tumor and non-tumor cells: *AluSq2* and *AluSx*. Both of these *Alus* belong to the intermediate-age subfamily *AluS*, whose members are thought to be inactive for retrotransposition [4]. *AluSq2* is antisense to the first intron of the gene NFIA and is expressed in five cell lines (H1-hESC, HeLa-S3, Hep G2, K562, NHEK) according to single-locus *Alu* expression profiling using ENCODE RNA-Seq data [34]. This *Alu* element lacks the internal A-rich motif A_6_TACA_5_, which is replaced by A_3_G. *AluSx* is antisense to the first intron of the AMFR gene and is expressed in NHEK cells [34]. A Control RNA sequence was amplified by PCR from the *Escherichia coli* LacZ gene, a sequence completely unrelated to any sequence in the human genome, while having a similar GC distribution and content as the two *Alus* (61% GC in *AluSq2*, 53% GC in *AluSx*, 61% GC in the Control sequence). For information on the genomic coordinates of the overexpressed *Alu* sequences and on the *Alus* and Control complete nucleotide sequence, see the Supplementary Materials. *AluSq2*, *AluSx*, and the Control RNA were inserted into a lentiviral vector under the control of the H1 promoter, an upstream-located (type 3) Pol III promoter that directs the expression of the RNase P RNA (H1 RNA) and is widely used for non-coding RNA (ncRNA) overexpression studies [35] (Figure 2). We chose this strategy because, despite the high expression levels generally observed in vitro for *Alu* elements carrying canonical A and B boxes, transfected *Alu* elements with the sole internal promoter usually give barely detectable transcription [36,37], while the H1 promoter was previously shown to drive high *Alu* transcription in transfected cells [38]. In order to select stable integrants and to directly monitor gene expression from the lentivirus vector, the puromycin resistance and eGFP genes, under the control of the PGK Pol II-dependent promoter, were present in the same vectors. Normal IMR90 human lung fibroblasts and HeLa cells were transformed with the lentivirus constructs carrying *AluSq2*, *AluSx*, and the Control RNA sequence, as well as empty vector, and stable integrants were isolated by puromycin selection. No major alteration in cell growth and morphology was observed during cell culture (see Figure S1, Supplementary Materials).

### 2.2. Validation of Alu Overexpression

**Figure 3:**
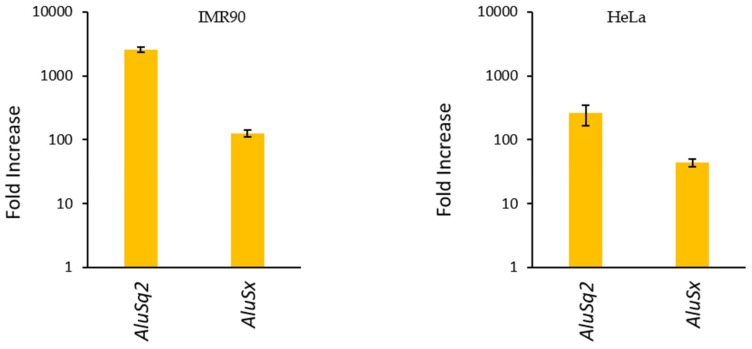
*Alu* overexpression from lentivirus vector stably inserted in the genome. The bar plots report the fold increase of *AluSq2* and *AluSx* expression in either (**left panel**) IMR90 or (**right panel**) HeLa cells that were stably transformed with a lentivirus vector carrying the corresponding *Alu*. The fold increase was derived from comparisons with cells transformed with the empty vector (background *Alu* expression). *Alu* RNA levels were quantified by RT-qPCR analysis, conducted with primers chosen to target unique sequence tracts within the *Alu* 3′ trailer region. In all measurements, gene expression levels were normalized to U1 gene expression, used as an internal standard.

The overexpression of *AluSq2*, *AluSx*, and Control RNA in stable integrant IMR90 and HeLa cells was evaluated by RT-qPCR. Since *Alu* elements are numerically abundant and repetitive, we used primers targeting the unique trailer regions of *AluSq2* and *AluSx* in order to unambiguously detect the transcripts of the transformed elements. As shown in Figure 3, in IMR90 the expression levels of both *Alus* were increased by 2 (*AluSx*) or 3 (*AluSq2*) orders of magnitude with respect to cells transformed with the empty vector, which were used as background control. Overexpression also occurred in HeLa cells, albeit to a lower extent (≈250-fold increase for *AluSq2*, ≈45-fold increase for *AluSx*). The lower increase in this case is likely due to higher background expression levels of the two *Alus* in this tumor cell line. *Alu* RNA levels were normalized with the expression of the control gene U1 snRNA, which is the most abundant snRNA with ≈1 × 10^6^ copies/cell [39].

### 2.3. Differential Gene Expression Analysis

**Figure 4:**
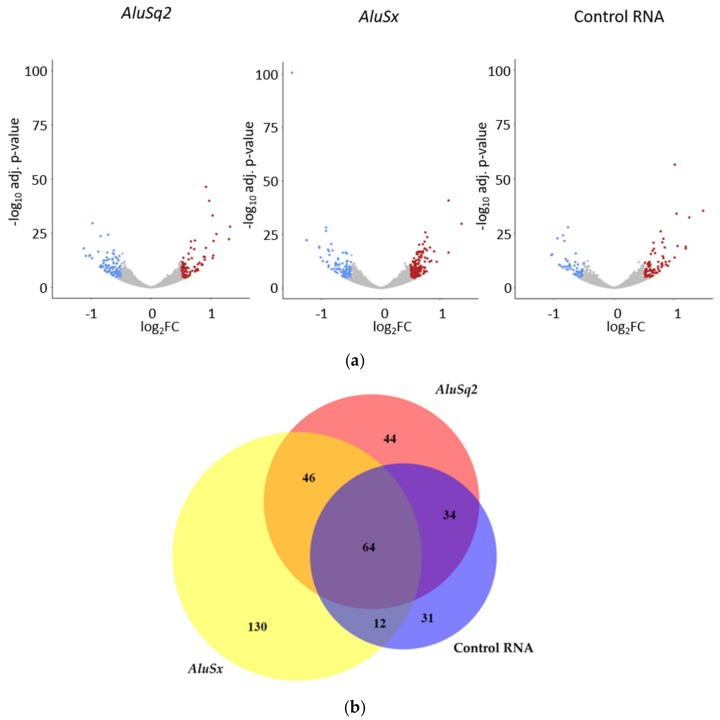
Differentially expressed genes in *Alu*/Control RNA-overexpressing cells. (**a**) Volcano plots showing the distribution of the differentially expressed genes in human fibroblasts overexpressing *AluSq2*, *AluSx*, or the Control RNA. Blue spots represent downregulated genes, red spots represent upregulated genes. (**b**) Venn diagram showing the intersection of genes that are differentially expressed in *AluSq2*, *AluSx*, and Control RNA-overexpressing cells.

To study *Alu*-dependent alteration of the protein-coding transcriptome, total RNA was extracted from *Alu*-overexpressing and Control-transformed IMR90 and HeLa cells and subjected to poly(A) enrichment for RNA-Seq analysis. Differential expression analysis (|log_2_FC|>0.5, adjusted *p*-value < 0.001) was then performed on RNA-Seq outputs by comparing cells overexpressing the gene of interest (*AluSq2*, *AluSx*, or Control RNA) with cells carrying the empty vector. In the case of IMR90, using DESeq2 [40] a total of 87 upregulated and 101 downregulated genes were found in *AluSq2*-overexpressing cells, 147 upregulated and 105 downregulated genes in *AluSx*-overexpressing cells, and 86 upregulated and 55 downregulated genes in cells stably overexpressing the Control RNA. The distribution of up- and downregulated genes in these samples is illustrated by the volcano plots in Figure 4a. The Venn diagram in Figure 4b shows the intersection of genes dysregulated in *AluSq2*-, *AluSx*- and Control RNA-overexpressing IMR90 cells. Remarkably, in contrast with IMR90 results, zero and two differentially expressed genes were detected in HeLa cells upon overexpression of *AluSq2* or *AluSx*, respectively (for a complete list of differentially expressed genes in IMR90 and HeLa cells, see Table S1, Supplementary Materials). 

**Figure 5:**
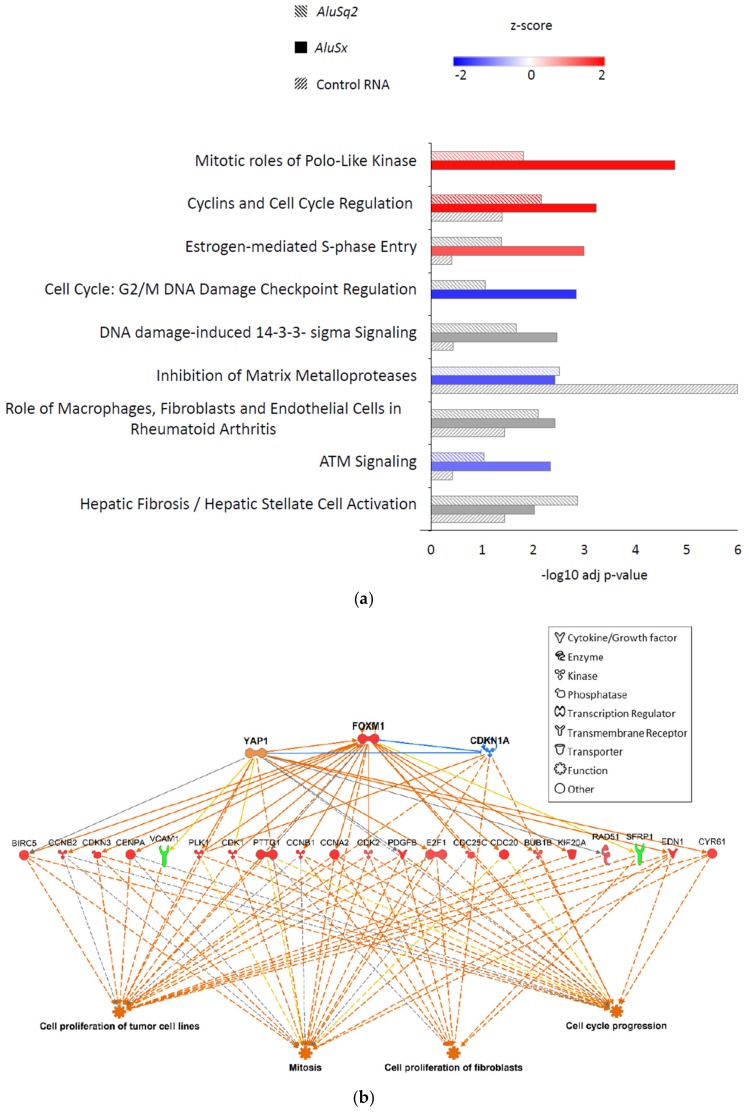

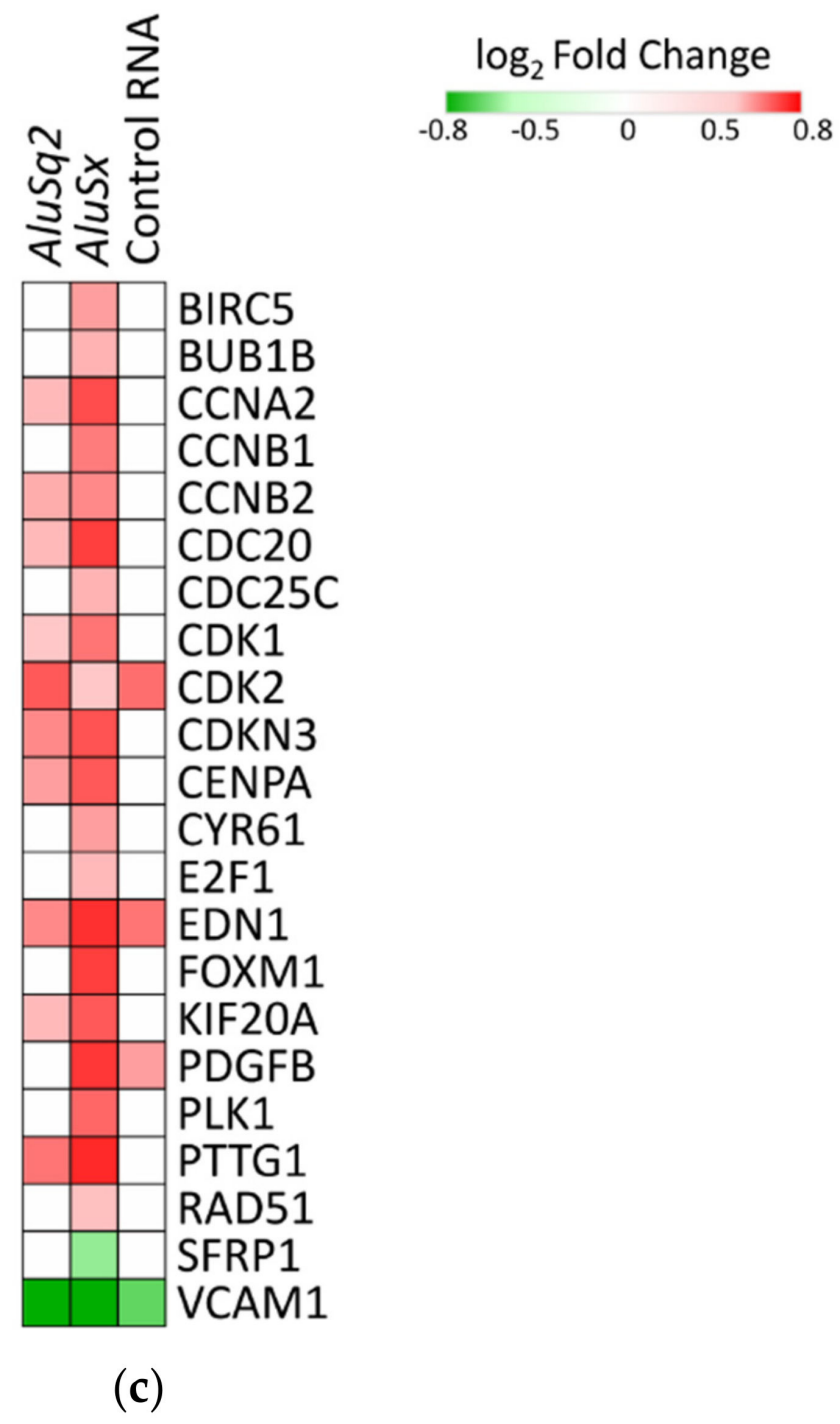
Dysregulation of cell cycle genes in *Alu*-overexpressing fibroblasts. (**a**) Pathways enriched in differential transcriptomes of *AluSq2*, *AluSx*, and Control RNA transformed fibroblasts as revealed by IPA analysis. Only pathways with *p*-value (BH correction) <0.01 in at least one comparison are shown and are ordered by *AluSx* vs. empty vector *p*-value. Z-score for activated or inhibited pathways is shown in red or blue, respectively. Grey bars: no predictions can be made. (**b**) Prediction of the upstream regulators that could modulate the expression of differentially expressed genes in *AluSx*-overexpressing fibroblasts and their effect on cell functions. Dysregulated genes are shown in the middle row as upregulated genes (red symbols) and downregulated genes (green symbols). Regulators are shown in the upper part of the figure. Blue indicates a predicted inhibition of the protein activity, while orange indicates a predicted activation. Blue lines indicate an inhibitory relationship, orange lines show an activating relationship, yellow lines indicate inconsistent relationship, while gray lines stand for no predicted effect. Continuous lines show direct interactions and dashed lines show indirect interactions (less than three passages). (**c**) Heatmap of differentially expressed genes shown in (**b**).

In order to understand which pathways are dysregulated in *Alu*- or Control-overexpressing IMR90 cells, we performed an enrichment analysis using the Ingenuity Pathway Analysis software (IPA, version number 48207413, Qiagen, Hilden, Germany). As shown in Figure 5a, several pathways related to the cell cycle (mitotic roles of Polo-like kinase, cyclins and cell cycle, estrogen-mediated S phase entry, G2/M DNA damage checkpoint) emerged as significantly enriched in *AluSq2*- and/or *AluSx*-overexpressing fibroblasts, with a general trend toward a higher significance in the case of *AluSx* with respect to *AluSq2*, but not in Control RNA datasets. In IMR90 cells overexpressing the Control RNA, only the pathway “Inhibition of Matrix Metalloproteases“ resulted in significantly enriched (adjusted *p*-value < 0.01). This effect was not investigated further. The *Alu*-specificity of the effect on cell cycle pathways excludes the possibility of gene expression perturbation trivially due to abnormal levels of an exogenous RNA sequence. Interestingly, pathways involved in a positive progression of cell cycle are detected as activated (red bars), whereas pathways that induce an arrest in cell cycle progression are detected as inhibited (blue bars), suggesting a role of *Alu* RNA in positively regulating cell cycle progression.

In order to predict which molecular species hypothetically influence the dysregulated expression patterns detected in Figure 5a, differentially expressed genes were analyzed for their enrichment in upstream regulators using the IPA software. This analysis is based on literature knowledge about the effects of regulators on gene expression. Therefore, any molecular species that can affect gene expression (i.e., transcription factors, microRNAs, kinases, compounds, or drugs) is taken into account. The literature-documented interaction between the upstream regulator and its target gene may have a direct role (direct interaction) in gene expression or can be involved in a cascade of upstream regulators (indirect interaction). Similarly, the downstream phenotypic effects can be inferred from the differentially expressed genes.

We focused our upstream regulator analysis on transcription factors and regulatory kinase systems known to be involved in the control of mitosis, and applied it to *AluSx*-dysregulated gene expression profiles. The transcriptional co-activator YAP1, the transcription factor FOXM1, and the cyclin-dependent kinase inhibitor 1A (CDKN1A) were predicted to participate in the control of the expression of dysregulated genes, as revealed by the upstream regulator analysis performed in IPA. Figure 5b shows a network describing the relationship among the most significant upstream regulators, the differentially expressed genes detected in *AluSx* overexpressing cells, and the inferred effects on cell proliferation of tumor cell lines, mitosis, cell proliferation of fibroblasts, and cell cycle progression. The dysregulated genes belong to CDC proteins, cyclins (CCNB2, CCNB1, CCN1), cyclin-dependent kinases (CDKs), proteins involved in mitotic spindle assembly and chromosome segregation (CENPA, KIF20A, PLK1, BUB1B), and soluble signaling molecules whose dysregulated expression is related to tumorigenesis (CYR61, EDN1, SFRP1, PDGFB). The case of FOXM1 is particularly interesting, since it is detected as an upregulated gene in our dataset as well as predicted as an upstream regulator.

Figure 5c shows the regulation of the differentially expressed genes that are predicted to be controlled by YAP1, FOXM1, and CDKN1A. Of note, eleven of these genes are also found dysregulated in *AluSq2*; in contrast, we could only detect four differentially expressed genes in the Control RNA-overexpressing cells. Overall, the results of IPA analyses support the hypothesis that *AluSx* modulates the transcription of cell cycle genes by acting through different mechanisms on key regulators such as YAP1, FOMX1, and CDKN1A.

### 2.4. *Alu* RNA Promotes IMR90 Cell Cycle Progression

**Figure 6:**
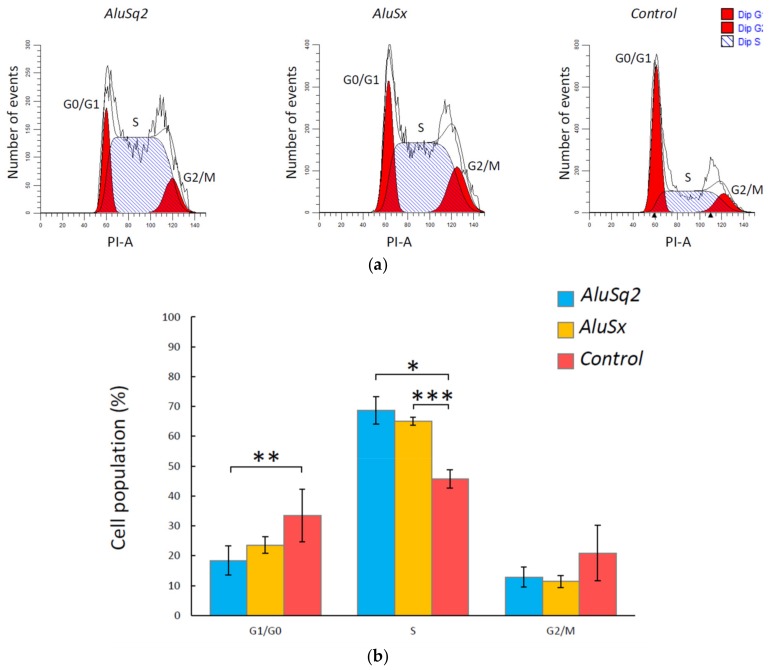
*AluSq2* and *AluSx* stimulate cell cycle progression of IMR90 cells. (**a**) FACS analysis of synchronized IMR90 cells after 24 h of serum re-feeding. ModFit LT analyses revealed an accumulation of *AluSq2*- and *AluSx*-overexpressing IMR90 cells in S phase. (**b**) Bar plot data are derived from at least four independent experiments obtained from two IMR90 cell cultures transformed with a lentivirus vector. * *p* < 0.05, ** *p* < 0.01, *** *p* < 0.0 01 compared to the Control.

We sought to experimentally assess whether cell cycle progression is affected by *Alu* RNA-dependent activation of cell cycle pathways. IMR90 cells overexpressing *AluSq2*, *AluSx*, or Control RNA were synchronized by serum starvation for 24 h, which resulted in 83 ± 2% of cells in G1 and 8% of cells in S phase, based on propidium iodide (PI) staining. After cell culturing in the starvation medium, serum was re-added and cell cycle distribution analysis was carried out after 24 h by flow cytometry. As shown in Figure 6a, reporting the results from several experiments carried out with two independently transformed IMR90 cell cultures, a significantly higher percentage of *Alu*-overexpressing cells were in S phase (and correspondingly, less were in G1) with respect to cells overexpressing the Control RNA. This effect was slightly more marked in the case of *AluSq2*, with 18% and 69% of cells in G1 and S phase, respectively, whereas 33% and 46% of cells in G1 and S phase, respectively, were observed in the Control RNA-overexpressing cells (Figure 6b). The data thus confirm that increased levels of *Alu* RNA promote cell cycle progression in IMR90 primary fibroblasts.

### 2.5. Location Analysis of *Alu* Elements at Differentially Expressed Loci

As a preliminary attempt to address the mechanism(s) of *Alu* RNA-dependent alteration of mRNA profiles, we checked for the presence of *Alu* elements in the promoter (2 kb upstream of the transcription start site (TSS)), in the 5′ UTR and in the 3′ UTR of differentially expressed protein-coding genes. A general trend towards an increase in the number of *Alus* in the 3′ UTR of downregulated genes and a decrease in the number of *Alus* in the 3′ UTR of upregulated genes was evident (Table 1). Compared to the total number of protein-coding genes, this effect was barely significant in the case of *AluSx*, while it was significant at the Fisher exact test for *AluSq2*-overexpressing IMR90 cells. When the orientation of *Alus* was taken into account, we could detect an increase of *Alu* sequences transcribed in an antisense orientation relative to up- and downregulated genes (*AluSq2*) or only to downregulated ones (*AluSx*) (Table 1). For a comprehensive list of all the *Alu* sequences in each differentially expressed gene, see Table S2, Supplementary Materials. 

To verify the possibility of a mechanistic connection of *Alu*-dependent upregulation with miRNA control, we searched for the presence of miRNA binding sites in *AluSq2* and *AluSx*. We were able to detect only the miRNA binding site for the sequence hsa-mir-619-5p, which was also predicted to target twelve and eleven differentially expressed genes in IMR90 cells overexpressing *AluSq2* and *AluSx*, respectively. In the latter one, the upregulated NCAPD2 is the only gene belonging to the cell cycle (Reactome pathway R-HSA-1640170) (Table S3, Supplementary Materials). 

## 3. Discussion

Our experiments support a positive role of increased *Alu* expression specifically in the proliferation of primary cells, but not of tumor cells. While the effects of *Alu* overexpression in primary differentiated cells have never been tested before, its effects on tumor cell proliferation have been addressed by several previous studies, with contrasting results. An early study based on the transient transfection of HeLa cells with *Alu*-carrying plasmids showed an *Alu*-dependent inhibition of cell proliferation, yet this effect could not be demonstrated to be caused by *Alu* RNA [29]. More recently, *Alu* and 7SL RNA transfection into MCF7 breast adenocarcinoma cells was found to entail a decrease in viability and an induction of pro-apoptotic changes [30]. In another recent study, *Alu* RNA transfection into the SW480 colorectal cancer cell line was instead found to be without effect on cell viability and cell cycle distribution, yet to induce epithelial-to-mesenchymal transition [27]. In other studies, more subtle effects of the expression of specific *Alus* were observed in human cells of embryonic origin. In particular, individual or specific subsets of *Alu* elements were shown to be activated in response to dioxin receptor AHR signaling [32] or to retinoic acid [31], with the production of *Alu*-derived transcripts inhibiting proliferation and promoting differentiation of human embryonic teratocarcinoma cells (NTera2) or human embryonic stem cells, probably through microRNA-like pathways. Taken together, the results of these studies delineate a complex scenario where cell lineage, differentiation state, and transformation degree are all relevant factors, perhaps together with the peculiar properties of individual *Alus*, in determining cell behavior in response to *Alu* overexpression. Within this framework, the most original contribution of our study consists in the use of primary cells (IMR90) and of stable transformation through lentiviral vectors to demonstrate that *Alu* RNAs can modulate the protein-coding transcriptome so as to promote cell cycling. IMR90 fibroblasts served recently as a good model for oncogenic transformation, also including early events in this process [41,42]. Therefore, the observation that *Alu* overexpression in these cells reprograms to some extent genome expression towards cell cycling appears as particularly relevant, as it suggests that increased *Alu* expression may be among the ways through which early oncogenic stimuli exert their effect. Along this line, the lack of effect of *Alu* overexpression on cell cycling or other pathways in HeLa cells may be due to the transformed state of these cells, with cell cycle and other pathways being already dysregulated as a consequence of this state. With this respect, our observations are in agreement with the recent observation that *Alu* RNA overexpression does not affect cell proliferation in another carcinoma cell line, SW480 [27]. A recent study reported that *Alu* transcription in human skin fibroblasts is suppressed in response to serum stimulation, via serum-dependent relocation to *Alu* of CGGBP1 followed by Pol III dislodging [43]. Since serum stimulation is expected to promote cell cycling, this study establishes a negative correlation between cell proliferation and *Alu* expression, in contrast with our observation that increased *Alu* RNA favors cell cycling. The study by Agarwal et al. [43], however, was based on serum starvation/stimulation, known to be complex signal sources producing wide reprogramming of epigenetic and transcriptional profiles, thus making it difficult to unravel the causal relationships between the different phenomena occurring in response to them [44]. Moreover, the causal involvement of *Alu* RNA in this study is based on the observation that CGGBP1 depletion entails increased *Alu* RNA levels as measured by RT-qPCR, and this is reflected by higher *Alu* RNA levels in quiescent cells than in serum-stimulated cells, again as revealed by RT-qPCR. From these observations, the authors infer that CGGBP1 relocation to *Alus* upon serum stimulation downregulates *Alu* expression. It should be noted, however (as the authors do), that the *Alu* RNA detected by this method could in large part be due to *Alu* sequences that are part of longer, RNA polymerase II-synthesized transcripts. It is thus uncertain whether the phenomena described in this study are causally related to general increase/decrease of genuine, Pol III-dependent *Alu* transcripts.

Given our experimental protocol, and based on the stable integration of an efficient *Alu* RNA-expressing cassette into the genome, the observed effects are most likely due to the activity *in trans* of *Alu* RNA (even though *in cis* effects of newly genome-integrated *Alu* cannot be formally excluded). A key unsolved question put forward by these observations is the mechanisms by which *Alu* RNAs lead to changes in the levels of specific mRNA subsets in IMR90 cells.

*Alu* RNA has previously been shown to function as a Pol II-interacting transcriptional repressor during the cellular heat shock response, thus contributing to downregulation of housekeeping genes (but not of other genes) under these conditions [25]. Our results are not inconsistent with the possibility that the overexpressed *Alu* elements in IMR90 cells could form *Alu* RNA-Pol II complexes, changing Pol II propensity to act at different subsets of promoters. 

As an alternative mechanism, mRNA upregulation could in principle be due to *Alu* RNA-dependent stabilization of mRNAs. Since regulatory ncRNAs that directly target mRNAs (such as miRNAs) generally cause mRNA destabilization, upregulation by *Alu* RNA could mechanistically rely on neutralization of mRNA-destabilizing ncRNAs (e.g., by miRNA sponging, [16]). Another possibility is related to the ability of free *Alu* RNA to interfere with Staufen-mediated mRNA decay, which is known to be favored by *Alu* sequences present in the 3′ UTR of mRNAs. For example, free *Alu* RNAs could saturate anti-*Alu* sequences carried by long non-coding RNAs that normally target mRNA for Staufen-mediated decay [45]. On the other hand, downregulation of protein-coding genes could be caused by RNA-duplexing of free *Alu* RNA with *Alu* sequences embedded in the 3′ UTR of mRNAs, inducing mRNA degradation mediated by the RNA-induced silencing complex (RISC), as suggested from our analyses in the case of downregulated genes in *AluSx*-overexpressing fibroblasts. 

As a factor further complicating the landscape of the *Alu* RNA mechanism of action, it is not known whether the effects that we observe on differentially expressed genes are due to full-length *Alu* RNA or to shorter processing products, nor whether important interactions of the overexpressed *Alu* RNA take place in the nucleus or in the cytoplasm. Difficulties in discussing this point derive from the fact that almost nothing is known about the biogenesis and processing pathways of *Alu* RNAs, except from the proposed role of Dicer1 in *Alu* RNA processing, which may result in the production of small regulatory RNAs potentially acting as mRNA destabilizers [31].

To what extent the *Alu* overexpression conditions we established are representative of naturally occurring physiological or pathological states is of course difficult to evaluate. However, increased levels of *Alu* RNA (or SINE-derived RNA in non-human mammals) are a common feature of cell response to different types of stress, including infection by viruses with the potential to drive cell transformation, such as adenovirus [46] and SV40 [47], and cancer progression [27]. Artificially increased *Alu* RNA levels in the absence of other oncogenic stimuli thus allow us to distinguish the contribution of these ncRNAs to the cell transformation process. Overall, the results of our analysis suggest that increased *Alu* RNA levels favor cell cycle progression thus contributing to sustained cell proliferation, which is an important factor for cancer development and progression [48].

## 4. Materials and Methods 

### 4.1. Cloning of Constructs

The *AluSq2* chr1:61057625-61057914 sequence was obtained by PCR amplification on human genomic DNA from saliva using the primer forward 5′-GCCCCAGGTGATCTCTACC-3′ and reverse 5′-GTCCTCGGAGCCGCTAATTT-3′, which anneal 200 nucleotides upstream of the transcription start site (TSS) and 100 nucleotides downstream of the Pol III terminator, respectively. The amplicon was cloned into the pGEM®-T easy vector using the pGEM®-T Easy Vector System kit (Promega, Madison, WI, USA). Subsequently, *AluSq2* was amplified from the pGEM®-T easy vector using the primer forward 5′-TAAATATAAAAGATCTGGCCAGGCGCTGTGGCT-3′, which introduces the restriction site *Bgl*II, (underlined) and the primer reverse 5′-AATTATTTTACTCGAGAAAAATGGCCACCACCGTTTCC-3′, which introduces the restriction site *Xho*I (underlined), in order to subclone *AluSq2* in the pSUPER.basic vector (OligoEngine™, Seattle, WA, USA). *AluSq2* was then subcloned from pSUPER.basic to pSUPER.GFP/neo (OligoEngine™) using the restriction enzymes *Bgl*II and *Xho*I, which allow the insertion of the fragment downstream of the H1 promoter (H1p). 

Similarly, the *AluSx*_chr16:56419511-56419806 sequence was first amplified from human genomic DNA using the primers forward 5′-CCCTTAACTTTTGTACCCTGAGC-3′ and reverse 5′-CACTCTGAACGGGGACAAGTA-3′. The primer forward anneals 183bp upstream of the *Alu* TSS and the primer reverse 75 bp downstream of the 6T *Alu* terminator. A second pair of primers (forward 5′-TAAATATAAAAGATCTGGCCAGGCGTGGTGG-3′ and reverse 5′-AATTATTTTACTCGAGAAAAAATGACTTGAAGCTTTGACAGCA-3′) was used to introduce the restriction sites for *Bgl*II and *Xho*I for cloning into the pSUPER.GFP/neo vector downstream of the H1 promoter (H1p).

The Control RNA sequence was amplified by PCR from the LacZ gene (*Escherichia coli* DH10 genomic DNA), which has the same distribution and GC content as the *Alu* sequences (61% GC *AluSq2*, 53% GC *AluSx*, 61% GC Control RNA sequence). Two rounds of PCR were performed: the first PCR was performed using the primers forward 5′-TAAATATAAAAGATCTGACCAGCGAATACCTGTTCC-3′ and reverse 5′- TTTTTTTTGGGGAGCGTCACACTGAG-3′, obtaining an amplicon that was used as template for a second PCR, which was performed using the same forward primer and the reverse primer 5′-AATTATTTTAAAGCTTTTTTTTTTTTTTTTTTTTGGGGAGCGTCAC-3′. In the last reverse primer, a poly(A) tail typical of *Alu* sequences was introduced. Moreover, the 3′end contains a *Hind*III site (underlined) that was used to subclone the Control RNA sequence in *AluSx*_pSUPER.GFP/neo vector where *AluSx* was excised using the restriction enzymes *Bgl*II and *Hind*III, in order to exploit the Pol III 6T natural terminator from *AluSx*. 

The sequences H1p-*AluSq2*, H1p-*AluSx*, H1p-Control RNA, and H1p-empty present in the pSUPER/GFP.neo expression plasmids were amplified by PCR with *Xba*I and *Bam*HI restriction sites added at the 5′ and 3′ ends, respectively, of the amplified fragment. Primers that annealed to the H1 sequence and *Alu*/Control RNA sequence/empty vector were used to amplify the fragment of interest. The primer forward 5′-GAGTTCTAGAGAACGCTGACGTCATCAACCC-3′ was used to start the amplification from the H1 promoter (the *Xba*I restriction site is underlined), while the primer reverse 5′-CCTCCGGATCCAAAAATGGCCCCACCGTTTCC-3′ annealed to the *AluSq2* sequence, 5′-CCTCCGGATCCAAAAAATGACTTGAAGCTTTG-3′ to *AluSx*, 5′-GAGTGGATCCAAAAAATGACTTGAAGC-3′ to Control RNA, 5′-CCTCCGGATCCGTCGACGGTATCGATAAGCTTAG-3′ to the empty vector (the *Bam*HI restriction site is underlined). The fragments were ligated in a 3rd generation lentiviral vector pRRL-MCS-PGK-GFP-IRES-Puro already digested with *Xba*I and *Bam*HI. The DNAs were isolated and the inserts sequenced. Clones containing the correct insert were amplified and purified using an Invitrogen PureLink™ HiPure Maxiprep Kit (Carlsbad, CA, USA).

Lentivirus-based vectors encoding *AluSq2*, *AluSx*, Control RNA as well as an empty vector were generated by transient cotransfection of 293T cells with a three-plasmid combination, as described previously, with slight modifications [49]. The construct pMD.G was used for the production of the VSV-G viral envelope in combination with the packaging constructs pMDLg/pRRE and pRSV–REV, whereas the pRRL constructs correspond to the different transfer vectors. Briefly, 100 mm dishes of non-confluent 293T cells were co-transfected with the four plasmids, by the CaPi-DNA coprecipitation method [50,51]. Conditioned medium was harvested 48 h later and passed through 0.45 mm filters.

Viral titer was determined by assessing viral p24 antigen concentration by ELISA (the Alliance^®^ HIV-I p24 ELISA Kit, PerkinElmer, Waltham, MA, USA) and hereafter expressed as μg of p24 equivalent units per milliliter.

### 4.2. Cell Lines and Lentiviral Vector Transduction

IMR90 and HeLa cells were purchased from the American Type Culture Collection (ATCC, Manassas, VA, USA). The cells were grown in Dulbecco’s Modified Eagle Medium (DMEM, purchased from ThermoFisher Scientific, Waltham, MA, USA) supplemented with 10% FBS (TCB, Tulare, CA, USA) and 100 U/mL penicillin/streptomycin (ThermoFisher Scientific, Waltham, MA, USA) and maintained in a humified atmosphere 5% CO_2_ at 37 °C. The day before the transduction, IMR90 and HeLa cells in the exponential phase of growth were plated in 6-well plates at 2 × 10^5^ cells per well. The next day, the medium was aspirated and the cells were incubated in 1 ml final volume with virus (0.86 μg/mL *AluSq2*, 0.75 μg/mL *AluSx*, 1.2 μg/mL Control RNA, 0.89 μg/mL empty vector) supplemented with 4 μg/mL protamine sulfate. The cells were incubated overnight in a humified atmosphere 5% CO_2_ at 37 °C and washed with fresh medium the next day. Stable integrants were selected by puromycin selection (2 mg/mL for IMR90 cells and 0.5 µg/mL for HeLa cells) starting 8 days after transformation. After 7 days of growth in selective medium, cells were expanded and grown for another 7–10 days before RNA extraction. All the transformations were performed in duplicate. 

Since cells were GFP fluorescent after more than one month of culture growing and we were able to detect the expression (by real-time PCR) of the insert controlled by the promoter H1 after puromycin selection, we could deduce that the lentivirus vector was efficiently inserted in the genome of IMR90 and HeLa cells.

### 4.3. RNA Extraction and Real-Time PCR

Total RNA was extracted from exponentially growing cells (passage 9 for IMR90 cells) using a Direct-zol™ RNA MiniPrep Plus kit (Zymo Research, Irvine, CA, USA) and the cDNA was synthesized using the SuperScript™ III Reverse Transcriptase (Thermo Fisher, Waltham, MA, USA). Real-Time PCR was performed using the PowerUp™ SYBR® Green Master Mix (Applied Biosystems, Foster City, CA, USA), the Applied Biosystems™ 7500 Real-Time PCR System (Applied Biosystems, Foster City, CA, USA), and a previously optimized pair of primers that anneal to the unique 3′ trailer sequence of *Alu* elements (primers forward 5′-AAGTGTCACCTCCCCATCTG-3′ and reverse 5′- ACCACCGTTTCCTGAGCTT-3′ for *AluSq2*, and the primer forward 5′-AATTCAACTATATTAAAACACTTCAGA-3′ and reverse 5′-GACTTGAAGCTTTGACAGCA-3′ for *AluSx*). The detection of the Control RNA was performed using the same primers employed for cloning. U1 snRNA was used as the internal normalization control. 

### 4.4. RNA-Seq Procedure and Data Analysis

One microgram of total RNA was used to prepare mRNA libraries using the TruSeq stranded mRNA library Preparation kit (Illumina, San Diego, CA, USA). A 50 base-pair single-end stranded sequencing was performed on a HiSeq4000 Sequencer (Illumina, San Diego, CA, USA). Read alignments to the GRCh38 human reference genome were performed using STAR [52]. HTSeq [53] and DESeq2 [40] were used for read counting and differential gene expression, respectively. Genes with |log_2_FC|≥0.5 and adjusted *p*-value ≤ 0.001 versus the empty vector were deemed as differentially expressed and visualized using the Volcano Plot workflow on the Galaxy web platform at the public server at https://usegalaxy.eu. Venn diagram was plotted using BioVenn [54] and enrichment analyses were performed with Ingenuity Pathway Analysis (IPA, QIAGEN Inc., Hilden, Germany). Only pathways with overlap *p*-values (BH-adjusted) < 0.01 were considered significantly enriched. Upstream regulator analysis in IPA was employed to identify the transcriptional regulators that could explain the observed gene expression changes. HeatMaps were visualized with Morpheus software (https://software.broadinstitute.org/morpheus).

### 4.5. Cell Proliferation Assay

To synchronize IMR90 cells overexpressing *Alus*/Control sequences, 5 × 10^5^ cells were seeded in 10 cm dishes in growth medium overnight. The next day, the cultures were rinsed with PBS and changed to serum-free medium. After serum starvation for 24 h, the cells were released into cell cycle by addition of 10% serum medium. After 24 h, the cells were trypsinized and fixed with 70% ethanol in PBS for at least 24 h at −20 °C. The day of FACS analysis, the cells were centrifuged and washed with PBS and the cell pellets were resuspended with propidium iodide (PI)-staining solution (100 ug/mL PI, 20 ug/mL RNAse). Samples were incubated in the dark for 15 min at room temperature and then analyzed using a BD FACSCelesta™ flow cytometer (BD Biosciences, San Jose, CA, USA). FACS analyses were performed in at least four replicates deriving from two independently transformed IMR90 cell lines, and the mean of PI fluorescence intensity was obtained from 20,000 cells. Cell cycle distributions were determined with ModFit LT™ software (version 5.0, Verity Software House, Topsham, ME, USA) and the statistical analyses were performed with a Student’s *t*-test or a Welch’s *t*-test.

### 4.6. Bioinformatic Analyses on Promoter, 5′ UTR, 3′ UTR, and miRNA Content of Differentially Expressed Genes

GENCODE v27 (Wellcome Trust Sanger Institute, Hinxton, UK) was used for the analysis of the content of *Alu* sequences in the promoter, 5′ UTR and 3′ UTR of differentially expressed genes. Only *Alu* sequences that overlapped for at least 100 bp were taken into account.

The sequences *AluSq2* and *AluSx* were interrogated using the miRBase database for the search of miRNA binding sites. The differentially expressed genes were then interrogated with the miRDB database to search potential binding sites for the sequence has-miR-619-5p.

## Figures and Tables

**Table 1:** Analysis of the presence of *Alu* sequences in the 3′ UTR of up- and downregulated genes in IMR90 cells overexpressing *AluSq2* or *AluSx*. Only the *Alu* sequences that were at least 100-bp long and fully overlapped with the 3′ UTR of target transcripts were considered in the analysis.

Sample	Genes with *Alu* in 3′ UTR	Total Number of Analyzed Genes	*p*-Value (Fisher exact test)	% of Genes with an *Alu* Element in Their 3′ UTR	% of Anti-sense *Alu* in Each Gene (Average)
Genome	4838	19,836	–	24.39	49.22
*AluSq2*_Up	9	87	0.0005	10.34	66.67
*AluSq2*_Down	37	101	0.0019	36.63	55.63
*AluSx*_Up	25	147	0.0082	17.01	30.00
*AluSx*_Down	28	105	0.0761	26.67	68.45

## Supplementary Materials

Supplementary Materials can be found at https://www.mdpi.com/1422-0067/20/13/3315/s1.

## Abbreviations

ncRNA	non-coding RNA
SINEs	short interspersed nuclear elements
L1	non-LTR retroelements LINE-1
UTR	untranslated region
FC	fold change
IPA	Ingenuity Pathway Analysis
H1p	H1 promoter
PI	propidium iodide
