# Supplementary material for: Alu RNA Modulates the Expression of Cell Cycle Genes in Human Fibroblasts
Source: Int J Mol Sci. 2019 Jul 5;20(13):3315. doi: 10.3390/ijms20133315 (PMC6651528; doi:10.3390/ijms20133315)
Supplement: Supplementary file 1 [file ijms-20-03315-s001.zip › Supplementary Figure S1.pdf]

Supplementary Figure S1

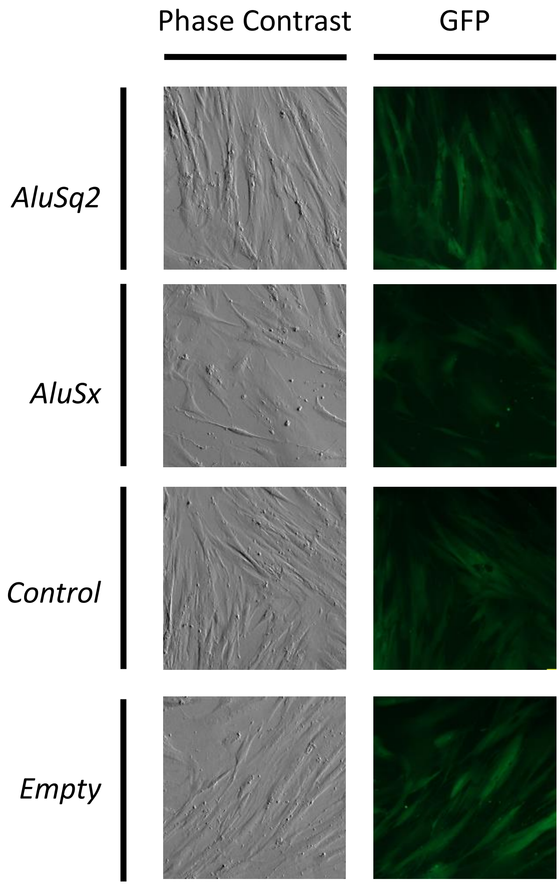

Figure S1. IMR90 cells overexpressing *AluSq2*, *AluSx*, Control or an empty lentivirus vector. Stable integrants were selected with puromycin treatment for two weeks. Only stably transformed cells were visualized as GFP-fluorescent cells. No big changes in cell morphology are observed comparing cells overexpressing *AluSq2*, *AluSx* or a control with cells transformed with an empty lentivirus vector.
