# Supplementary material for: Alu RNA Modulates the Expression of Cell Cycle Genes in Human Fibroblasts
Source: Int J Mol Sci. 2019 Jul 5;20(13):3315. doi: 10.3390/ijms20133315 (PMC6651528; doi:10.3390/ijms20133315)
Supplement: Supplementary file 1 [file ijms-20-03315-s001.zip › Supplementary Information.docx]

**Supplementary Information**

The genomic coordinates of *Alu* sequences correspond to the annotated element. The 3’ trailer is underlined. In the Control RNA sequence the portion deriving form *AluSx* is underlined.

| **Name of *Alu*** | *AluSq2* |
| --- | --- |
| **Genomic coordinates** | chr1:61057625-61057914 |
| **Genome assembly** | GRCh38/hg38 |
| ***AluSq2* sequence:** |  |

>AluSq2_chr1:61057625-61057914

GGCCAGGCGCTGTGGCTCACGCCTGTAATCCCAACACTTTGGGAGGCCGAGGCGAGTGGATCACCTGAGGTCAGGAGTTCGCGACCAGCCTGACCCACATGGTGAAACCCCGTCTCTACTAAAGTTAGCCAGACGTGGTGGCCGGCGCCTGTGATCTCAGCTACTCGGGAGGCTGAGGCAGGAGAATCGCTTGTACCCGGGAGGCGAGGTTGCAGTGAGCCGAGATCGCGCCATTGCAGTCCAGCCTGGGCGACAAGAGCGAAACTCCGTCTAAAAAAAAAAAAAAAAAAGTGTCACCTCCCCATCTGCAAAGGTCTGGCCTCCTGAAAGCTCAGGAAACGGTGGGGCCATTTTT

| **Name of *Alu*** | *AluSx* |
| --- | --- |
| **Genomic coordinates** | chr16:56419511-56419806 |
| **Genome assembly** | GRCh38/hg38 |
| ***AluSx* sequence:** |  |

>AluSx_ chr16:56419511-56419806

GGCCAGGCGTGGTGGCTCATGCCTGTAATCTCAGCACTTTGGGAGGCCAAGGTTAGTGGATCACCTGAGGTCAGCAGTTCAAGACCAGCCTGGCCAACATGGTGAAATCCCGTGTCTACTAAAAATACAAAAAATTAGCTGGGCATGGTGGTGCACACCTGTAATCTCAGCTACTTGGGTGGCTGAGGCAGGAGAATTGCTTGAACCCAGGAGGCGGAGATTGCAGTGAGCCGAGATTGTGCCATTGCACTCCAGCCTGGGCGACAGAGAAAGACTCTGACTCAAAAAAAAAAAAATTCAACTATATTAAAACACTTCAGAATGTTTCTCATAGCTATAGTGCTGTCAAAGCTTCAAGTCATTTTTT

**Control RNA sequence:**

>control_RNA

GACCAGCGAATACCTGTTCCGTCATAGCGATAACGAGCTCCTGCACTGGATGGTGGCGCTGGATGGTAAGCCGCTGGCAAGCGGTGAAGTGCCTCTGGATGTCGCTCCACAAGGTAAACAGTTGATTGAACTGCCTGAACTACCGCAGCCGGAGAGCGCCGGGCAACTCTGGCTCACAGTACGCGTAGTGCAACCGAACGCGACCGCATGGTCAGAAGCCGGGCACATCAGCGCCTGGCAGCAGTGGCGTCTGGCGGAAAACCTCAGTGTGACGCTCCCCGCTTCAAGTCATTTTTT
